# A Temperate Life

**Published:** 1860-06

**Authors:** 


*A Temperate Life.*—Gen. Cass, now in his seventy-eighth year, is said to have never tasted any kind of intoxicating liquor. Through all the vicissitudes of his arduous and eventful life, he has avoided the deceptive aid of alcoholic stimulation. If such a career as his, attended with many toils and temptations, has not needed artificial stimulus, there can be little excuse for the most of those who are attracted to that in which "discontent seeks for comfort; cowardice for courage; sadness for joy; and all find *ruin*."—*Medical and Surgical Reporter.*

